# Association between Serum Ferritin and Blood Lipids: Influence of Diabetes and hs-CRP Levels

**DOI:** 10.1155/2020/4138696

**Published:** 2020-03-24

**Authors:** Lianlong Yu, Jingyi Yan, Qian Zhang, Hong Lin, Lichao Zhu, Qiangqiang Liu, Changsheng Zhao

**Affiliations:** ^1^Institution of Food and Nutrition, Shandong Center for Disease Control and Prevention, Ji'nan, Shandong, China; ^2^Law Enforcement and Supervision Bureau of Shandong Provincial Health Commission, Ji'nan, Shandong, China; ^3^Qianfoshan Hospital, Ji'nan, Shandong, China; ^4^Department of Pediatric Surgery, Shandong Provincial Hospital Affiliated to Shandong University, Ji'nan, Shandong, China; ^5^Department of General Practice, Qilu Hospital of Shandong University, Ji'nan, Shandong, China; ^6^Department of Nutriology, The Second Hospital of Shandong University, Ji'nan, Shandong, China

## Abstract

This study is aimed at exploring the relationship between serum ferritin and blood lipids and the influence of diabetes and different hs-CRP levels. A total of 8163 subjects were analyzed. Participators were classified according to serum ferritin, diabetes, and two hs-CRP levels. Blood lipids were determined using standardized methods and conditions. Except for HDL-C, there was a significant increase in blood lipids in the progressive ferritin group with normal hs-CRP levels (*P* < 0.05). But HDL-C was just the opposite (*P* < 0.0001). In nondiabetic patients, TG, TC, and LDL-C were significantly elevated in the progressive ferritin group (*P* < 0.05). And, HDL-C was just the opposite (*P* < 0.05). The generalized linear model and the parsimonious model showed that serum TG was positively correlated with ferritin, and LDL-C was negatively correlated with ferritin (*P* < 0.05). But the correlation between LDL-C and ferritin was broken (*P* > 0.05). After a sufficient adjustment, there was a positive correlation between serum TG and ferritin and a negative correlation between LDL-C and ferritin. Nonetheless, a negative correlation between LDL-C and ferritin is influenced by diabetes frailly. And, there was no change of relationship between lipids and ferritin in different hs-CRP levels. We found a real relationship between ferritin and lipids after sufficient adjustment for confounders.

## 1. Introduction

In mammals, ferritin should be a way for cells to store iron, rather than transporting iron, whereas the measurement of serum ferritin (SF) levels is widely used for iron status indicators [1]. In addition, SF levels can be significantly increased to cope with inflammation and/or multiple diseases [2]. Ferritin not only functions as a clinical biomarker for the evaluation of iron status but also plays an important role in energy metabolism disorder. Elevated SF levels are closely related to chronic diseases such as type 2 diabetes (T2D) and cardiovascular disease. Correlation analyses revealed a negative correlation between the levels of SF and high-density lipoprotein cholesterol (HDL-C), while positive correlations existed between the levels of SF and triglyceride (TG), total cholesterol (TC), and low-density lipoprotein cholesterol (LDL-C) [3].

Meanwhile, some of the studies showed that elevated serum high-sensitive C-reactive protein (hs-CRP) is related to abnormal lipid metabolism [4]. Ferritin levels were related (Pearson's) to average IL-6 levels (*r* = 0.1845; *P* = 0.002) and hs-CRP levels (*r* = 0.1175; *P* = 0.04) in a previous research. Vascular experts can consider measuring ferritin levels while assessing lipid and hs-CRP levels. Ferritin levels, inflammatory cytokines, hs-CRP, and mortality are related statistics showing that iron-induced oxidative stress may be associated with inflammatory response to PAD [5].

However, limited studies considered the relationship between ferritin and lipids in serum and the influence of hs-CRP or type 2 diabetes. The purpose of our study was to investigate the changes of the relationship between SF and lipids influenced by diabetes and the level of hs-CRP.

## 2. Research Design and Methods

### 2.1. Study Design

The data of our research was based on the China Health and Nutrition Survey (CHNS). This project is a national cross-sectional survey, whose contents were surveying the Chinese health status. The data in our research was selected from this survey database in 2009. This survey was designed using a stratified multistage cluster random process to take samples from 9 provinces of China, including Liaoning, Hunan, Henan, Shandong, Jiangsu, Heilongjiang, Guizhou, Hubei, and Guangxi. All subjects voluntarily attended in this survey with informed consent. And also, this survey was approved by an institutional review board of the University of China Center for Disease Control and Prevention and the University of North Carolina at Chapel Hill.

### 2.2. Study Population

Participants aged 18 years or above were included in this research. Basic information such as age, gender, activity level, region, and dietary behavior were collected. Finally, there were 8163 subjects organized in the surveys (2009). These subjects were investigated using questionnaire to cover a series of related biochemical markers, health factors, and nutrition measurement [6].

Our study was an analysis of 8163 subjects included in the 2009 organized survey, which included complete information on lipid, ferritin, T2D, and hs-CRP measurements. All subjects in this study underwent the same examination.

### 2.3. Measurements and Definitions

Subjects over the age of 18 went to community service centers, where fasting blood samples collected by trained physicians were taken to local centers for disease control. Special efforts are made during planned morning or weekend visits to avoid missing children in boarding schools and migrant workers.

Management of the unified training by the collaborating teams was organized before the survey, and quality control was overseen via the tablet software and quality control member during their visits. The blood pressure of the right arm, including systolic and diastolic blood pressure, was measured using a suitable cuff size mercury sphygmomanometer. Blood pressure was collected three times after 10 minutes of quiet rest. The interval between the two measurements was more than 30 seconds. Testing of ferritin, lipids, hs-CRP, and hemoglobin A1c (HbA1c) were to be completed at the Ministry of Health laboratory of the China-Japan Friendship Hospital.

Fasting blood samples were collected by venipuncture. The tests for glucose and HbA1c were immediately taken. Plasma and serum samples were then frozen and stored at -86°C for laboratory analysis. All samples were analyzed at the National Central Laboratory in Beijing (Medical Laboratory Certification ISO 15189: 2007) with strict quality control. We focus on blood biomarkers for the risk of heart disease associated with diabetes.

Height, weight, and waist circumference were measured by trained inspectors in accordance with the standard procedures of the World Health Organization and measured to an accuracy of 0.1 kg. Height, weight, and waist measurements were taken at the same location and follow the same procedure for each study visit. Central obesity was defined by WC > 90 cm for men and >80 cm for women [7, 8].

Subjects were classified according to type 2 diabetes (yes/no) and hs-CRP levels (0-3.0 mg/L group, ≥3.0 mg/L group) initially and SF (below 10.0 ng/mL, 10.0-299.9 ng/mL, and over 300.0 ng/mL) afterwards followed the classification of CHNS. Other information gathered from the survey include sex, age, physical examination, dyslipidemia, dietary factors, and use of antidiabetic drugs.

### 2.4. Statistical Methods

*P* values < 0.05 were considered statistically significant. Comparison between ferritin groups in basic information and clinical characteristics were used by the *χ*^2^ test for categorical variables and ANOVA for continuous variables. In particular, we calculated and tested the differences in blood lipids among the three ferritin groups and among type 2 diabetes (yes/no) and hs-CRP level (0-3.0 mg/L group, ≥3.0 mg/L group) subgroups.

A generalized linear model of the association between blood lipids (TC, TG, HDL-C, and LDL-C) and ferritin (three groups) was built adjusting for age (continuous), sex, BMI (continuous), waist circumference (continuous), diabetes (yes/no), hs-CRP (continuous), HbA1c (%), insulin injection (yes/no), carbohydrate intake (continuous), urea (continuous), uric acid (continuous), apolipoprotein A-1 (continuous), apolipoprotein B (continuous), lipoprotein (continuous), creatinine (continuous), insulin (continuous), antidiabetic drug treatment (yes/no), blood pressure (continuous), energy intake (continuous), fat intake (continuous), and protein intake (continuous).

Separate adjusted models were also built for subjects whether suffering from type 2 diabetes or not. And we also performed more parsimonious models as a sensitivity analysis, excluding adjustment for some variables dealing with possible collinearity among some covariates.

## 3. Results

Our study involved 8163 subjects with an average age of 50.9 years and 46.7% were males. 8.3% of the subjects were with type 2 diabetes (Table 1) and 17.1% with high hs-CRP level. Blood lipid averages (TG, TC, HDL-C, and LDL-C) were 1.33 mmol/L, 4.59 mmol/L, 1.38 mmol/L, and 2.82 mmol/L.

As shown in Table 1, 85.2% of the subjects had a normal SF level, 5.3% had a low level (≤10 ng/mL), and 9.6% had a high level (≥300.0 ng/mL). As depicted in Table 1, subjects with a higher level of SF featured significantly higher blood pressure (*P* < 0.0001), older age (*P* < 0.0001), higher waist circumference (*P* < 0.0001), higher BMI (*P* < 0.0001), and higher dietary intake (energy, fat, protein, and carbohydrate) (*P* < 0.01).

**Figure 1:**
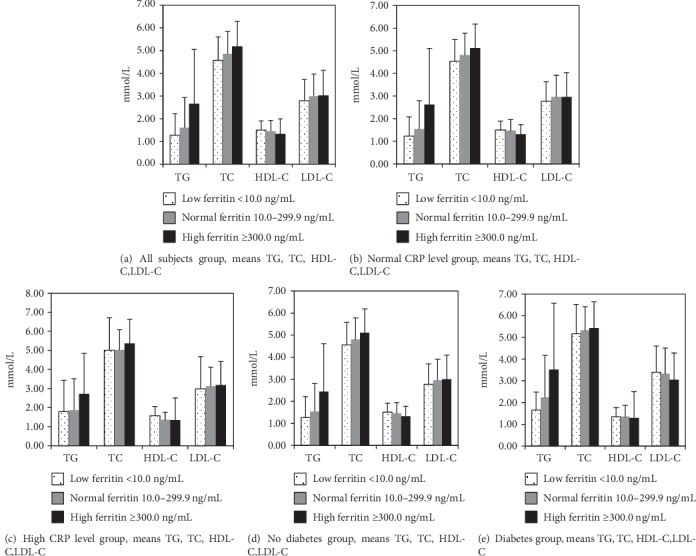
TG, TC, HDL-C, and LDL-C were significantly higher among subjects with a high CRP level than among those with a normal CRP level (*P* < 0.05). A significant increase of TG, TC, and LDL-C across progressive ferritin groups was observed in subjects without diabetes (*P* < 0.05). However, no significant change of HDL-C and LDL-C across progressive ferritin groups was observed in subjects with diabetes (*P* > 0.05). And also, TG, TC, HDL-C, and LDL-C were significantly higher among subjects with diabetes than among those without diabetes (*P* < 0.05). (a) All-subject group, means TG, TC, HDL-C, and LDL-C. (b) Normal CRP level group, means TG, TC, HDL-C, and LDL-C. (c) High CRP level group, means TG, TC, HDL-C, and LDL-C. (d) No diabetes group, means TG, TC, HDL-C, and LDL-C. (e) Diabetes group, means TG, TC, HDL-C, and LDL-C.

As the SF levels get higher, there was an increasing prevalence of diabetes, obesity, and central obesity and a significantly higher biochemical indicator (*P* < 0.01), but this did not include lipoprotein and apolipoprotein A-1 values (Figures 1(a)–1(e)).

Table 2 and Figures 1(b) and 1(c) reveal blood lipid values in the overall sample classified by the hs-CRP and ferritin levels. Except for HDL-C, across progressive ferritin groups (from low ferritin level ≤ 10 ng/mL, normal level = 10.0 − 299.9 ng/mL, to high level ≥ 300.0 ng/mL), increasing trends of blood lipids in the overall sample were noted (*P* < 0.05). This fact was also happening in subjects with a normal level of hs-CRP. However, there were no significant changes of HDL-C and LDL-C across progressive ferritin groups in a subgroup with a higher hs-CRP level (*P* > 0.05). TG, TC, and LDL-C levels were significantly higher in the subgroup with a higher hs-CRP level than in the normal subgroup level (*P* < 0.0001). However, HDL was significantly higher among subjects with a normal hs-CRP level than among those with a higher hs-CRP level (*P* < 0.0001).

Table 3 and Figures 1(d) and 1(e) depicted the status of subjects whether having type 2 diabetes or not. In a subgroup of subjects without type 2 diabetes, a significantly higher level of TG, TC, and LDL-C across progressive ferritin groups was observed (*P* < 0.05). And a significant decrease in HDL-C along with increasing ferritin was detected in subjects without diabetes (*P* < 0.05). Nevertheless, no significant changes of TG and HDL-C were found in subjects with diabetes along with increasing ferritin (*P* > 0.05). And also, no significant changes of TG, TC, HDL-C, and LDL-C along with increasing ferritin were detected in subjects with diabetes and a high hs-CRP level (*P* > 0.05).

### 3.1. Multivariable Analysis

The generalized linear model presented that TG was significantly higher in the subgroup with ferritin ≥ 300.0 ng/mL than those in subgroups with lower levels (*P* < 0.05), after full adjustment of demographic characteristics, dietary factors, lifestyles, and clinic biomarkers (including blood pressure, diabetes, insulin, blood glucose, insulin injection, antidiabetic drugs treatment, uric acid, urea, apolipoprotein A-1, creatinine, apolipoprotein B, and lipoprotein). However, LDL-C was significantly lower in a subgroup of ferritin ≥ 300.0 ng/mL than those in subgroups with lower levels (*P* < 0.05). According to the 95% confidence interval, there were no differences between subjects with the SF level < 10.0 ng/mL and those with a level of 10.0-299.9 ng/mL in TG and LDL-C (*P* > 0.05).

There was a significantly positive correlation between serum TG and ferritin and a negative correlation between LDL-C and ferritin after adjustment.

Multivariable models for TC, HDL-C, and LDL-C were unable to get statistical significance in the type 2 diabetes subgroup. The results of the parsimonious model were quite similar to the aforementioned general pattern (Table 4). Therefore, this phenomenon was revealed to be quite similar to the type 2 diabetes subgroup, neither in a generalized linear model nor in a parsimonious model. The relationship between lipids and ferritin did not change in different hs-CRP levels (not depicted in Table 4).

## 4. Discussion

Diabetes is often associated with dyslipidemia. Our research is no exception. In this research, diabetes disturbed the association between the SF level and lipids (TG, TC, and HDL-C) in the higher hs-CRP level (≥3.0 mg/L). And also, diabetes reversed the positive correlation between ferritin and LDL-C in the lower CRP level (0-3.0 mg/L). Previous studies [9, 10] found that dyslipidemia is one of the main risk factors for type 2 diabetes. Routine indicators of lipids, including TG, TC, HDL-C, and LDL-C, have been well documented in relation to the development of type 2 diabetes [11, 12]. At the same time, type 2 diabetes is closely related to lipase activity [13].

This study found that SF was positively correlated with BMI, waist circumference, insulin, blood glucose, TG, TC, LDL-C, and other indicators (*P* < 0.05), except for HDL-C. Meanwhile, after a sufficient adjustment for confounders, increasing ferritin concentrations were related with higher TG only in lipids. Similarly, in Han et al.'s study, elevated ferritin concentrations were closely associated with higher lipids, insulin, glucose, BMI, and waist circumference (*P* < 0.0001) [14]. Our study supported this view. And also in some studies, increasing ferritin levels have been displayed to be associated with lipemia [15], elevated blood glucose, and fasting insulin [16], forecasting incident type 2 diabetes in prospective studies [17, 18]. These results were so similar to our research findings.

In our study, there was a significantly higher prevalence of obesity, central obesity, diabetes, and elevated biochemical indicator (*P* < 0.01), along with an increasing SF level, except for apolipoprotein A-1 and lipoprotein values. In a previous study, elevated levels of ferritin have been known as a feature of type 2 diabetes. The relationship between type 2 diabetes and iron levels is complex. Insulin activates the iron upload and accelerates ferritin synthesis, and contrarily iron influences the insulin reduction of glucose production from the liver [19, 20].

The EPIC-Norfolk prospective study explained that SF is an important and independent predictor of diabetes development [21]. Increased iron storage is also positively correlated with the incidence of metabolic syndrome (MetS) and insulin resistance in American adults [22], as well as body fat distribution, waist/hip ratio, and obesity in Mexican-Americans aged 20-49 years [23]. Increased iron storage may interfere with the extraction of hepatic insulin, leading to excess insulin in peripheral blood [24]. Studies have shown that iron regulates the role of insulin in healthy people and in people with type 2 diabetes [25]. In addition, iron regulates iron reduction, insulin secretion, endothelial dysfunction, and metabolic control in people with type 2 diabetes [25]. There are also benefits. Studies have also confirmed the relationship between metabolic disorders and elevated iron reserves in the Chinese population [19, 26, 27]. Shi et al.'s research shows that iron intake and iron status are independently associated with diabetes risk in Chinese women but not in men. The effect of gender on the prevalence of metabolic diseases is reported to vary among different populations [28]. There are significant differences between women and men in the role of insulin, susceptibility to insulin resistance, and response to stimuli. In essence, women are more likely to develop insulin resistance than men. Factors such as sex hormones, the environment, and lifestyles increase or improve the “genetic” disadvantages of women, and these factors may also be genetically determined [29]. These research findings were similar to our study (Table 1).

In addition, elevated levels of ferritin in the blood are associated with an increased risk of type 2 diabetes in the elderly, an increased risk of diabetes in adults in northern China, and a higher risk of diabetes in north Chinese adults [27, 30]. Although SF levels vary widely between men and women and the relationship between women and men in China vary from diabetes to diabetes [26], scientific evidence predicts that elevated SF levels may have an effect on IR and type 2 diabetes due to increased iron reserves in the body or may be affected by multiple inflammatory diseases [31, 32].

In our study, a higher hs-CRP level and T2DM disturbed the relationship between SF and lipids. Subclinical inflammation with activated cytokines is also a key feature of this disease.

For the production of cytokines, inflammatory reaction is accompanied by the generation of acute phase proteins, mainly CRP [33]. Speculated CRP causes serine phosphorylation in the insulin receptor, weakening the latter's phosphatidyl inositol 3-kinase activation and leading to the development of insulin resistance [34].

In this paper, with the increase of the SF level, the hs-CRP level increased obviously (*P* < 0.01). High ferritin levels without significant iron overload may affect glucose homeostasis, leading to insulin resistance and inflammatory changes, such as elevated CRP levels [35]. In other researches, mean hs-CRP levels vary from one corresponding to ferritin levels [36]. In men, it is associated with age, smoking, alcohol, cardiovascular history, body mass index (BMI), waist circumference, blood pressure, blood lipids, CRP, adiponectin, alanine aminotransferase (ALT), and glutamine. After the adjustment of peptidase (GGT), the elevated SF is associated with diabetes [37].

Our study confirms that elevated serum hs-CRP is associated with abnormal lipid metabolism in adults. This connection was also confirmed in a given population in previous studies [38, 39]. In Quebec, in children ages 9 years, 14 years, and 16 years, elevated CRP levels were found to be independently associated with worsening lipid status (high TG and low HDL-C concentrations). [40]

Surprisingly, serum TG was positively correlated with ferritin after adequate adjustment of confounding factors, and LDL-C was negatively correlated with ferritin. However, LDL-C is negatively correlated with ferritin and is susceptible to diabetes. At the same time, there was no change in the relationship between lipid and ferritin at different hs-CRP levels.

Our study has some limitations. First, we have corrected some confounding factors, but our study lacks data on blood *n*‐3 levels, which may affect blood lipids. Second, further research is needed to explore the exact cause of the observed metabolic changes in T2D effects. Third, we lack data on iron intake from the diet. Fourth, cross-sectional analysis of the data does not allow for a causal assessment of the relationship being studied. Fifth, our study lacks data on lipoprotein lipase levels, which may explore basal metabolism. Sixth, despite the use of detailed adjustments and stratification, we recognize that animal experiments are necessary to prove results. We hope that future research will address this area.

In summary, the above contradictory results that confuse us are due to their inadequate adjustment of confounding factors. At the same time, limited studies examined the relationship between SF and blood lipids affected by type 2 diabetes and hs-CRP after adequate adjustment of confounding factors. Thus, we found the real relationship between ferritin and lipids after a sufficient adjustment for confounders.

In short, there was a positive correlation between serum TG and ferritin and a negative correlation between LDL-C and ferritin. But the negative correlation between LDL-C and ferritin is influenced by diabetes frailly. This may be due to the role of ferritin in lipoprotein lipase activity, as described by Ryan et al. [41]. At the same time, type 2 diabetes is closely related to lipase activity [13].

## Figures and Tables

**Table 1:** Sample characteristics according to ferritin status (mean values and standard deviations; numbers and percentages).

	Total	Low ferritin < 10.0 ng/mL	Normal ferritin = 10.0 − 299.9 ng/mL	High ferritin ≥ 300.0 ng/mL	*P* value
Variables	(*n* = 8163)	(*n* = 431)	(*n* = 6951)	(*n* = 781)	
Age (years)	50.9 ± 15.1	41.8 ± 12.0	51.4 ± 15.2	51.5 ± 14.0	<0.0001
≥60 years	2275 (27.9)	33 (7.7)	2033 (29.3)	209 (26.8)	<0.0001
Risk factors					
Systolic pressure (mmHg)	124.9 ± 19.0	117.0 ± 15.9	125.1 ± 19.2	127.7 ± 17.7	<0.0001
Diastolic pressure (mmHg)	80.3 ± 11.2	76.3 ± 10.1	80.1 ± 11.2	83.2 ± 11.4	<0.0001
Myocardial infarction	81 (0.99)	2 (0.47)	75 (1.08)	4 (0.51)	0.6651
Cardiovascular stroke	114 (1.4)	2 (0.47)	100 (1.44)	12 (1.54)	0.2080
BMI (kg/m^2^)	23.4 ± 3.5	22.6 ± 3.2	23.3 ± 3.5	24.6 ± 3.3	<0.0001
Obesity (BMI ≥ 30 kg/m^2^)	338 (4.14)	7 (1.62)	278 (4.00)	53 (6.79)	<0.0001
Waist circumference (cm)					
Men	84.4 ± 10.2	82.1 ± 9.1	83.8 ± 10.2	87.7 ± 10.0	<0.0001
Women	81.3 ± 10.3	77.9 ± 9.6	81.4 ± 10.3	87.3 ± 10.1	<0.0001
Central obesity	3135 (38.4)	154 (35.7)	2634 (37.9)	347 (44.4)	0.0005
Diabetes (%)	679 (8.32)	12 (2.78)	519 (7.47)	148 (18.95)	<0.0001
Diabetes (men) (%)	327 (8.63)	6 (11.76)	215 (6.91)	106 (16.85)	<0.0001
Diabetes (women) (%)	342 (7.92)	6 (1.59)	295 (7.78)	41 (27.33)	<0.0001
High CRP level (0-3.0 mg/L)	1394 (17.08)	32 (7.42)	1185 (17.05)	177 (22.66)	<0.0001
Dietary factors					
Energy intake (kcal/day)	2141.3 ± 658.7	2011.3 ± 569.0	2133.6 ± 661.1	2265.3 ± 665.4	<0.0001
Fat intake (g/day)	75.4 ± 39.7	68.9 ± 30.0	75.1 ± 40.0	81.3 ± 41.1	<0.0001
Protein intake (g/day)	65.8 ± 22.9	62.1 ± 21.0	65.4 ± 22.8	71.0 ± 23.5	<0.0001
Carbohydrate intake (g/day)	295.1 ± 101.4	284.3 ± 95.3	294.5 ± 101.4	303.5 ± 104.3	0.006
Analytical values					
HbA1c (%)	5.6 ± 0.9	5.5 ± 0.6	5.6 ± 0.9	6.0 ± 1.4	<0.0001
Urea (mmol/L)	5.5 ± 1.6	4.7 ± 1.4	5.5 ± 1.6	5.8 ± 1.6	<0.0001
Uric acid (mg/dL)	308.3 ± 106.3	239.3 ± 70.8	304.7 ± 99.9	379.1 ± 136.2	<0.0001
Apolipoprotein A-1 (g/L)	1.2 ± 0.4	1.2 ± 0.3	1.2 ± 0.4	1.1 ± 0.3	<0.0001
Apolipoprotein B (g/L)	0.9 ± 0.3	0.8 ± 0.3	0.9 ± 0.3	1.0 ± 0.3	<0.0001
Lipoprotein (mg/dL)	15.6 ± 22.9	15.3 ± 21.9	15.8 ± 23.1	13.9 ± 20.2	0.0799
Creatinine (*μ*mol/L)	87.4 ± 22.5	78.9 ± 16.5	87.1 ± 20.3	94.8 ± 37.2	<0.0001
Insulin (*μ*IU/mL)	14.4 ± 22.4	11.6 ± 8.9	14.3 ± 23.1	16.9 ± 21.2	0.0003
Insulin (men) (*μ*IU/mL)	14.6 ± 23.7	10.0 ± 6.96	14.4 ± 25.0	16.1 ± 16.7	0.1149
Insulin (women) (*μ*IU/mL)	14.1 ± 20.6	11.8 ± 9.1	14.1 ± 21.5	17.7 ± 17.2	0.0113
Antidiabetic drugs					
Oral drugs	182 (2.2)	0 (0)	146 (2.10)	36 (4.61)	<0.0001
CRP (mg/L)	2.6 ± 9.0	1.3 ± 2.9	2.5 ± 8.6	4.0 ± 13.8	<0.0001
Insulin injection	51 (0.62)	0 (0.0)	42 (0.60)	9 (1.15)	0.0125

Data are given as the mean ± SD or *n* (%).

**Table 2:** Blood lipid values according to hs-CRP levels and ferritin status (mean values and standard deviations).

	Total							Normal CRP = 0 − 3.0 mg/L							High CRP ≥ 3.0 mg/L						
	Low ferritin < 10.0 ng/mL		Normal ferritin = 10.0 − 299.9 ng/mL		High ferritin ≥ 300.0 ng/mL			Low ferritin < 10.0 ng/mL		Normal ferritin10.0 − 299.9 ng/mL		High ferritin ≥ 300.0 ng/mL			Low ferritin < 10.0 ng/mL		Normal ferritin = 10.0 − 299.9 ng/mL		High ferritin ≥ 300.0 ng/mL		
	Mean	SD	Mean	SD	Mean	SD	*P*	Mean	SD	Mean	SD	Mean	SD	*P*	Mean	SD	Mean	SD	Mean	SD	*P*
TG (mmol/L)	1.28	0.94	1.60	1.33	2.64	2.41	<0.0001	1.23	0.85	1.54	1.25	2.62	2.48	<0.0001	1.80	1.63	1.87	1.64	2.72	2.12	<0.0001
TC (mmol/L)	4.57	1.03	4.85	0.99	5.17	1.11	<0.0001	4.54	0.95	4.81	0.97	5.12	1.06	<0.0001	5.01	1.71	5.03	1.05	5.36	1.27	0.0011
HDL-C (mmol/L)	1.51	0.40	1.45	0.48	1.32	0.67	<0.0001	1.50	0.39	1.47	0.49	1.31	0.43	<0.0001	1.58	0.49	1.36	0.40	1.35	1.16	0.0851
LDL-C (mmol/L)	2.79	0.94	2.99	0.97	3.01	1.13	0.0001	2.77	0.86	2.96	0.96	2.96	1.08	0.0006	2.98	1.69	3.12	1.00	3.18	1.24	0.5893

Data are given as the mean and SD for continuous variables.

**Table 3:** Blood lipid values according to hs-CRP levels and ferritin status in patients without diabetes and in patients with diabetes.

	Total							Normal CRP = 0 − 3.0 mg/L							High CRP ≥ 3.0 mg/L						
	Low ferritin < 10.0 ng/mL		Normal ferritin = 10.0 − 299.9 ng/mL		High ferritin ≥ 300.0 ng/mL			Low ferritin < 10.0 ng/mL		Normal ferritin = 10.0 − 299.9 ng/mL		High ferritin ≥ 300.0 ng/mL			Low ferritin < 10.0 ng/mL		Normal ferritin = 10.0 − 299.9 ng/mL		High ferritin ≥ 300.0 ng/mL		
	Mean	SD	Mean	SD	Mean	SD	*P*	Mean	SD	Mean	SD	Mean	SD	*P*	Mean	SD	Mean	SD	Mean	SD	*P*
Patients without diabetes																					
TG (mmol/L)	1.27	0.94	1.54	1.26	2.44	2.17	<0.0001	1.22	0.85	1.51	1.23	2.44	2.20	<0.0001	1.81	1.68	1.74	1.38	2.43	2.06	<0.0001
TC (mmol/L)	4.56	1.02	4.81	0.97	5.11	1.08	<0.0001	4.52	0.93	4.78	0.95	5.07	1.02	<0.0001	5.00	1.77	4.99	1.03	5.28	1.28	0.0203
HDL-C (mmol/L)	1.51	0.40	1.46	0.48	1.33	0.45	<0.0001	1.51	0.39	1.47	0.49	1.33	0.44	<0.0001	1.61	0.50	1.38	0.41	1.32	0.47	0.0032
LDL-C (mmol/L)	2.77	0.93	2.96	0.95	3.00	1.10	<0.0001	2.76	0.84	2.94	0.94	2.97	1.04	0.0008	2.94	1.73	3.11	0.98	3.16	1.28	0.5896
Patients with diabetes																					
TG (mmol/L)	1.65	0.83	2.24	1.93	3.50	3.08	<0.0001	1.65	0.88	2.06	1.50	3.58	3.53	<0.0001	1.69	0.78	2.62	2.56	3.37	2.13	0.1216
TC (mmol/L)	5.17	1.35	5.32	1.10	5.42	1.23	0.5712	5.15	1.48	5.34	1.06	5.35	1.22	0.8664	5.23	0.57	5.28	1.17	5.54	1.24	0.3529
HDL-C (mmol/L)	1.35	0.42	1.35	0.52	1.28	1.23	0.5552	1.38	0.45	1.40	0.59	1.19	0.34	0.0041	1.22	0.24	1.25	0.34	1.43	1.99	0.5313
LDL-C (mmol/L)	3.40	1.21	3.33	1.18	3.04	1.25	0.0305	3.35	1.29	3.39	1.23	2.93	1.29	0.0072	3.63	0.95	3.22	1.08	3.23	1.17	0.8753

Data are given as the mean and SD for continuous variables.

**Table 4:** Generalized linear model of the association between blood lipids and ferritin according to diabetes status.

	Ferritin groups						*P* value
	<10.0 ng/mL		10.0-299.9 ng/mL		≥300.0 ng/mL		
	Mean	95% CI	Mean	95% CI	Mean	95% CI	
Full model							
Total							
TG (mmol/L)	1.79	1.65-1.93	1.70	1.64-1.77	2.11	2.00-2.22	<0.0001
TC (mmol/L)	4.85	4.79-4.91	4.87	4.84-4.90	4.92	4.87-4.97	0.0833
HDL-C (mmol/L)	1.43	1.38-1.47	1.42	1.40-1.45	1.40	1.37-1.44	0.47
LDL-C (mmol/L)	2.93	2.84-3.00	2.97	2.94-3.00	2.85	2.80-2.90	<0.0001
Subjects without diabetes							
TG (mmol/L)	1.62	1.50-1.74	1.54	1.50-1.58	1.91	1.82-2.01	<0.0001
TC (mmol/L)	4.81	4.75-4.86	4.84	4.83-4.86	4.89	4.84-4.93	0.0906
HDL-C (mmol/L)	1.45	1.41-1.49	1.45	1.44-1.47	1.44	1.41-1.48	0.7287
LDL-C (mmol/L)	2.95	2.89-3.00	3.00	2.98-3.01	2.88	2.83-2.92	<0.0001
Subjects with diabetes							
TG (mmol/L)	2.05	0.96-3.14	2.38	2.19-2.56	2.86	2.56-3.16	0.0095
TC (mmol/L)	5.53	5.09-5.97	5.32	5.25-5.40	5.41	5.28-5.53	0.3157
HDL-C (mmol/L)	1.47	1.27-1.67	1.32	1.29-1.36	1.30	1.24-1.35	0.1891
LDL-C (mmol/L)	3.58	3.01-4.15	3.33	3.23-3.43	3.26	3.10-3.42	0.4631
^∗^Parsimonious model							
Total							
TG (mmol/L)	1.80	1.67-1.94	1.70	1.64-1.76	2.11	2.01-2.21	<0.0001
TC (mmol/L)	4.86	4.80-4.91	4.89	4.87-4.89	4.93	4.88-4.97	0.0915
HDL-C (mmol/L)	1.43	1.38-1.47	1.43	1.41-1.45	1.41	1.38-1.44	0.4836
LDL-C (mmol/L)	2.93	2.87-2.99	2.96	2.94-2.99	2.84	2.80-2.87	<0.0001
Subjects without diabetes							
TG (mmol/L)	1.63	1.51-1.75	1.54	1.51-1.57	1.91	1.82-2.01	<0.0001
TC (mmol/L)	4.81	4.75-4.87	4.85	4.83-4.86	4.89	4.84-4.93	0.1103
HDL-C (mmol/L)	1.45	1.41-1.49	1.45	1.44-1.46	1.43	1.40-1.47	0.6878
LDL-C (mmol/L)	2.94	2.88-2.99	2.99	2.97-3.00	2.87	2.82-2.91	<0.0001
Subjects with diabetes							
TG (mmol/L)	1.97	1.03-2.92	2.36	2.22-2.50	2.88	2.61-3.15	0.0026
TC (mmol/L)	5.47	5.06-5.87	5.34	5.28-2.40	5.40	5.29-5.51	0.5575
HDL-C (mmol/L)	1.42	0.99-1.84	1.32	1.26-1.38	1.37	1.25-1.49	0.7288
LDL-C (mmol/L)	3.45	3.02-3.88	3.29	3.22-3.35	3.17	3.05-3.30	0.195

Models based on lifestyle, demographic characteristics, clinical variables, and dietary factors were adjusted. ^∗^The parsimonious model is adjusted to the same variables as the full model, except for some variables that deal with possible colinearity between certain covariates.

## Data Availability

The data used to support the findings of this study are available from the corresponding author upon request.
